# Telecommunication Wavelength-Band Single-Photon Emission from Single Large InAs Quantum Dots Nucleated on Low-Density Seed Quantum Dots

**DOI:** 10.1186/s11671-016-1597-0

**Published:** 2016-08-30

**Authors:** Ze-Sheng Chen, Ben Ma, Xiang-Jun Shang, Yu He, Li-Chun Zhang, Hai-Qiao Ni, Jin-Liang Wang, Zhi-Chuan Niu

**Affiliations:** 1 School of Physics and Nuclear Energy Engineering, Beihang University, Beijing, 100191 China; 2 State Key Laboratory for Superlattices and Microstructures, Institute of Semiconductors, Chinese Academy of Science, Beijing, 100083 China; 3 Hefei National Laboratory for Physical Sciences at Microscale and Department of Modern Physics, University of Science and Technology of China, Hefei, Anhui 230026 China; 4 CAS Center for Excellence and Synergetic Innovation Center in Quantum Information and Quantum Physics, University of Science and Technology of China, Hefei, Anhui 230026 China

**Keywords:** Bilayer quantum dot, Telecommunication wavelength, Single-photon source

## Abstract

Single-photon emission in the telecommunication wavelength band is realized with self-assembled strain-coupled bilayer InAs quantum dots (QDs) embedded in a planar microcavity on GaAs substrate. Low-density large QDs in the upper layer active for ~1.3 μm emission are fabricated by precisely controlling the indium deposition amount and applying a gradient indium flux in both QD layers. Time-resolved photoluminescence (PL) intensity suggested that the radiative lifetime of their exciton emission is 1.5~1.6 ns. The second-order correlation function of *g*^2^(0) < 0.5 which demonstrates a pure single-photon emission.

## Background

Real single-photon sources (SPSs) at telecommunication wavelength are very important for optical fiber-based quantum information that is practical for security application [1–3]. Semiconductor single quantum dots (QDs) are the best candidates to emit true single photons. In recent decades, InAs QDs [4, 5] have attracted great interest in quantum information [6, 7] and quantum computation [8]. Being compatible with GaAs/Al(Ga)As distributed Bragg reflectors (DBRs) to form a planar microcavity to enhance QD emission, GaAs-based InAs QDs have demonstrated high-intensity single-photon emission in wavelength <1000 nm [9]. However, it is difficult to obtain 1.3 μm or longer-wavelength single-photon emission on them, which was limited by QD material itself. Although InAs/InP QDs can emit single photons in a 1.55-μm wavelength range [10, 11], it is difficult to combine them with a high-Q DBR cavity. To extend the emission wavelength of InAs/GaAs QDs to telecommunication wavelength, several methods have been developed, such as asymmetric InGaAs/GaAs dot-in-well (DWELL) structure [12], 30 % In-content InGaAs capping layer [13], strain engineering of QDs [14], metamorphic structures [15], and vertically coupled QDs, i.e., bilayer QDs (BQDs) [16–18] where active QDs are formed on seed QDs capped with InGaAs. The BQD structure is advantageous on the growth since their growth parameters, such as growth rate, spacer layer thickness, indium deposition amount, and growth temperature can be precisely controlled. High-density BQDs have been grown to realize laser diodes at ~1.5 μm [17, 19]. However, few reports have forced on the growth of low-density BQDs for telecommunication-band single-photon emission. In fact, to realize low-density BQDs, the density and distribution of both the seed QDs and the active QDs and their interlayer strain coupling must be well controlled, which is more difficult than the growth of the traditional single-layer low-density QDs in wavelength <1000 nm.

In this paper, low-density active QDs with emission wavelength at 1.3 μm are realized on low-density seed QDs by well utilizing the interlayer strain coupling and precisely controlling the gradient indium deposition amount in seed QD layer and active QD layer on a non-rotating substrate. These BQDs are embedded inside a GaAs 1-λ cavity in the DBR structure to enhance QD emission in the normal direction. The second-order correlation function measurement demonstrates nice single-photon emission. This is the first time to apply this material system on telecommunication-band single-photon emission. The emission intensity of such single QDs could be improved by optimizing the growth parameters, e.g., the growth temperature of QDs.

## Methods

**Fig. 1:**
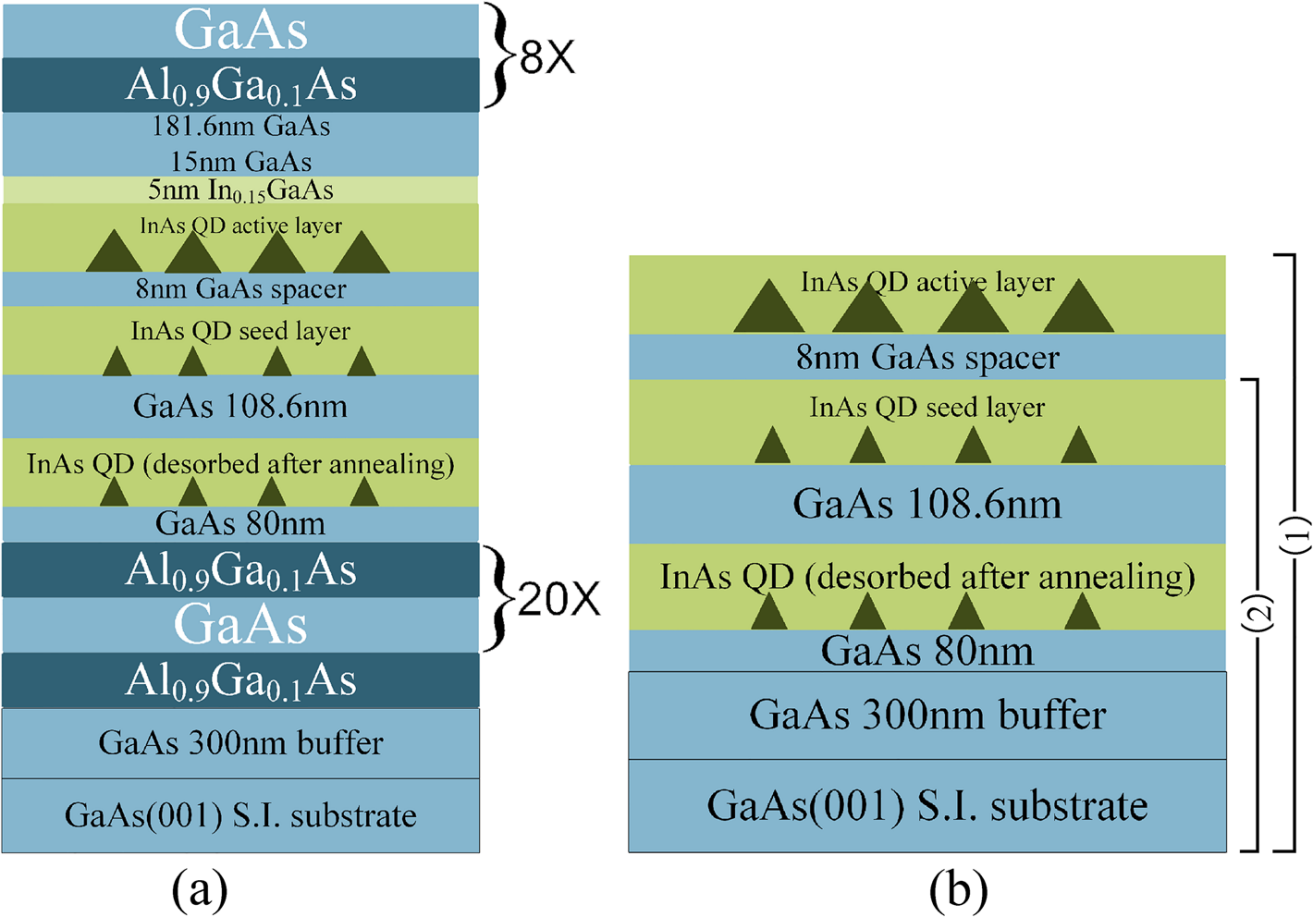
Schematic structures of sample A, BQDs in a DBR cavity (**a**) and sample B, uncapped BQDs (*1*) or uncapped seed QDs (*2*) (**b**)

The samples were grown by solid source molecular beam epitaxy (MBE; VEECO Gen930 system) on semi-insulating (100) GaAs substrates. The structure of sample A is shown in Fig. 1a. After a 300-nm-thick GaAs buffer layer growth at 580 °C, a planar 1-λ GaAs microcavity sandwiched between 20.5 pairs of the bottom and eight pairs of the top Al_0.9_Ga_0.1_As (113.7 nm)/GaAs (98.6 nm) DBR was grown, whose quality factor (Q), as characterized later, is ≈200. The integration into the DBR cavity increases the extraction efficiency of photon emission from a single active QD. In fact, without DBR, there is no micro-photoluminescence (μPL) signal detected in our experiment. The BQD structure capped with 5 nm In_0.15_Ga_0.85_As was embedded at the cavity antinode to maximize the cavity enhancement [17, 20, 21]. The QD growth is detailed as follows: on the 80-nm GaAs above the bottom DBR, a sacrificed InAs quantum dot (SQD) layer was deposited at 540 °C by using a very low growth rate of 0.005 ML s^−1^ and a periodic growth method with a 5-s interruption after each 20-s deposition of indium, under As_2_-stabilized condition. The critical coverage (*θ*_c_) for island formation was monitored by reflection high-energy electron-diffraction (RHEED). As RHEED monitored, the *θ*_c_ was 2.2 ML. After it, the SQD growth was stopped immediately and the substrate was annealed at 670 °C for 10 min to desorb SQDs completely [22]. Then, a 108.6-nm GaAs was deposited at 580 °C and the substrate temperature was cooled down to 540 °C to grow seed QDs. The substrate rotation was stopped to build a gradient indium flux on the substrate and form density-graded seed QDs. The growth rate and deposition method is the same as that of SQDs but with a sub-*θ*_c_ indium coverage. After 5 s of growth interruption, the GaAs spacer was grown at 540 °C immediately. It was in moderate thickness (i.e., 8 nm), allowing a capping of seed QDs and a residual strain field on them (as nucleation sites) in favor of vertically aligned active QD formation [20, 23]. A subsequent annealing at 610 °C for 10 min was used to remove the intrinsic defects and flattening the surface [18, 24–26] before deposition of the second QD layer. The substrate temperature was cooled down to 480 °C, and 2.0 ML of InAs coverage was deposited in a growth rate of 0.02 ML s^−1^ to form the active QDs. After a 5-s growth interruption, a 5-nm In_0.15_Ga_0.85_As strain-reducing layer (SRL) [20] was grown immediately to maintain QD height for longer-wavelength QD emission [23, 24]. The high substrate temperature and low growth rate of seed QDs increase the migration of indium atoms to form low-density islands [16, 27, 28], while the relatively lower substrate temperature and higher growth rate for active QDs reduce their migration and intermixing with Ga and enable a prior and rapid nucleation of single large QDs on seed QDs driven by strain, in a high optical quality [24, 29–31]. On the SRL, a 15-nm GaAs was capped in the same temperature and the rotation of substrate was resumed for the following steps. Finally, the substrate temperature raised to 580 °C to grow the top 181.6-nm GaAs and the top eight pairs of DBR. Sample A with the full cavity structure was used for optical measurements. As shown in Fig. 1b, sample B (reference sample) with uncapped BQDs (1), or uncapped seed QDs (2), grown in the same condition but with a *θ*_c_ indium coverage in seed QD layer, was used for morphology in atomic force microscopy (AFM). μPL spectroscopy was performed using the confocal microscopy technique with fiber-coupled input and output, excited by a 632.8-nm He-Ne laser, and detected by a liquid nitrogen-cooled CCD detector in the spectrometer. A high numerical aperture objective (×100, NA = 0.55) focuses the laser on the sample, in a diameter of ~2 μm, and enables an effective collection of QD luminescence in this micro-region. The spectral line of single QD emission was filtered for further measurement. Time-resolved PL measurement used a pulsed semiconductor laser (*λ* 780 nm, repetition rate 80 MHz, pulse width 3 ps, power 3.6 μW) as excitation, multi-channel superconducting nanowire single-photon detectors (SNSPD; time resolution 120 ps) for detection, and a Hydra Harp 400 module for time-correlated single photon counting (TCSPC), to investigate the radiative lifetime of the spectral line. To check its single-photon property, the second-order correlation measurement was done in Hanbury-Brown-Twiss (HBT) scheme [27, 32–34] by splitting the signal into two parts with a 50:50 beam splitter in free space and detecting them with two channels of SNSPD separately in fiber coupling, and analyzing the coincidence of both SNSPD outputs with TCSPC.

## Results and Discussion

**Fig. 2:**
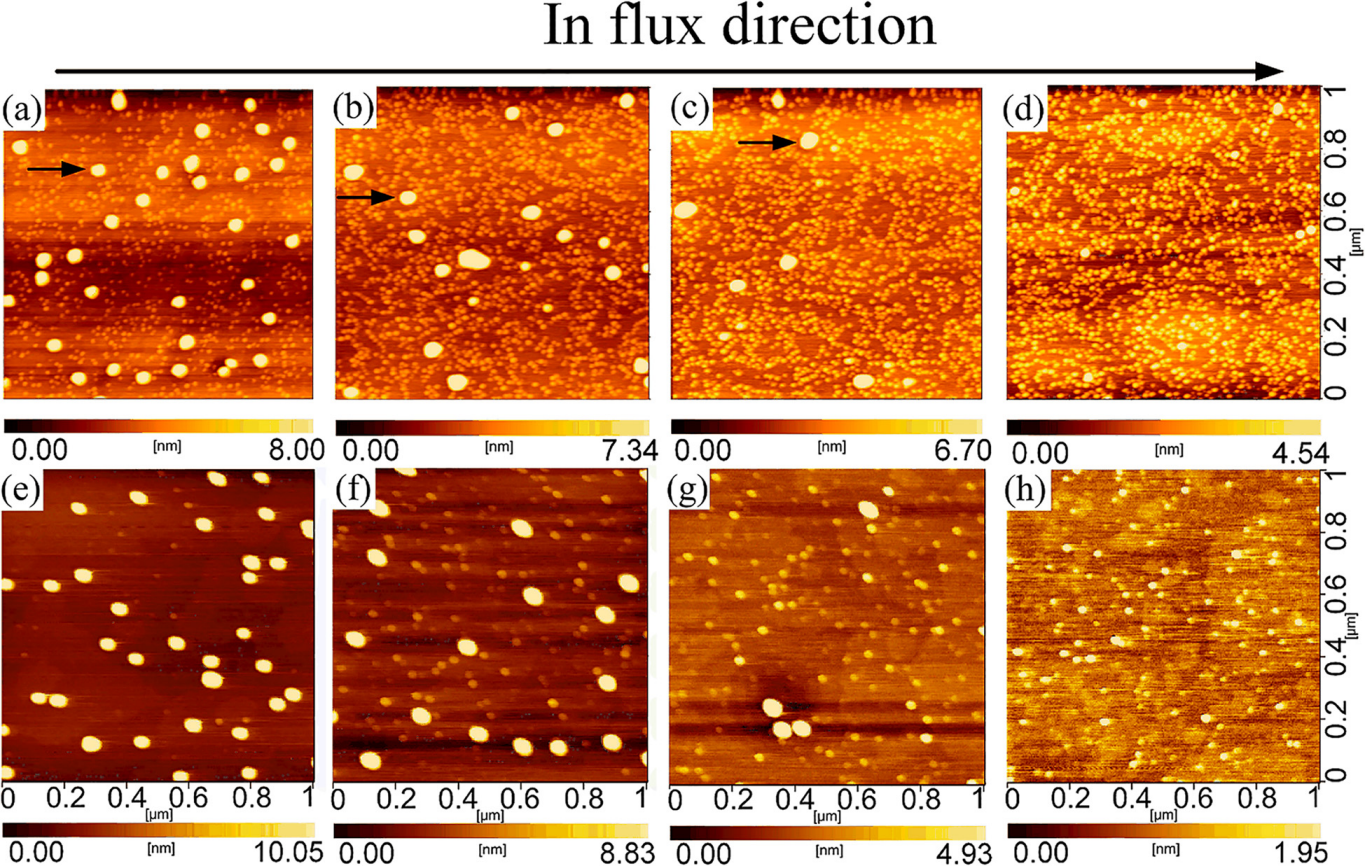
1 × 1 μm^2^ AFM images of uncapped active QDs (**a**–**d**) and uncapped seed QDs (**e**–**h**) along the direction of In flux gradient. *Small black arrows* indicate active QDs for 1.3 μm emission

Figure 2a–d shows the AFM images of active QDs along the direction of In flux gradient. Figure 2e–h shows the AFM images of seed QDs, correspondingly. The gradient In flux formed density-graded large QDs [35], among dense small QDs, i.e., bimodal QD size distribution. As In deposition amount decreases along the In flux direction, the large QDs evolve from high density (∼3.5 × 10^9^ cm^−2^ in Fig. 2a and ~1.8 × 10^9^ cm^−2^ in Fig. 2b) to low density (~6 × 10^8^ cm^−2^ in Fig. 2c), and finally no large QDs (Fig. 2d). In contrast, the density of small QDs in active QD layer increased as the indium deposition decreases, from about 8.8 × 10^10^ cm^−2^ in Fig. 2a to ~1.4 × 10^11^, ~1.7 × 10^11^, and ~2 × 10^11^ cm^−2^ in Fig. 2b–d, respectively. It reflects the collection of small QDs into a larger one during QD growth. In seed QD layer, the density of small QDs is much lower; the density of large QDs reduces from 3.4 × 10^9^ cm^−2^ in Fig. 2e to 1.8 × 10^9^, 4 × 10^8^ cm^−2^, and zero in Fig. 2f–h, respectively, similar to the large QDs in the active QD layer. It suggests that the large active QDs are grown on the large seed QDs whose strain field is strong enough to build nucleation sites, enabling a prior QD nucleation, while the dense small QDs in the active layer nucleate due to the low growth temperature and high growth rate that reduced surface diffusion of In atoms [36]. Therefore, the gradient In flux is an efficient way to get low-density BQDs. However, their density is still too high to realize a single QD in a micro-region for single-photon emission. So, in sample A for optical measurement, a sub-*θ*_c_ indium coverage in seed layer was used to reduce the density of active QDs.

**Fig. 3:**
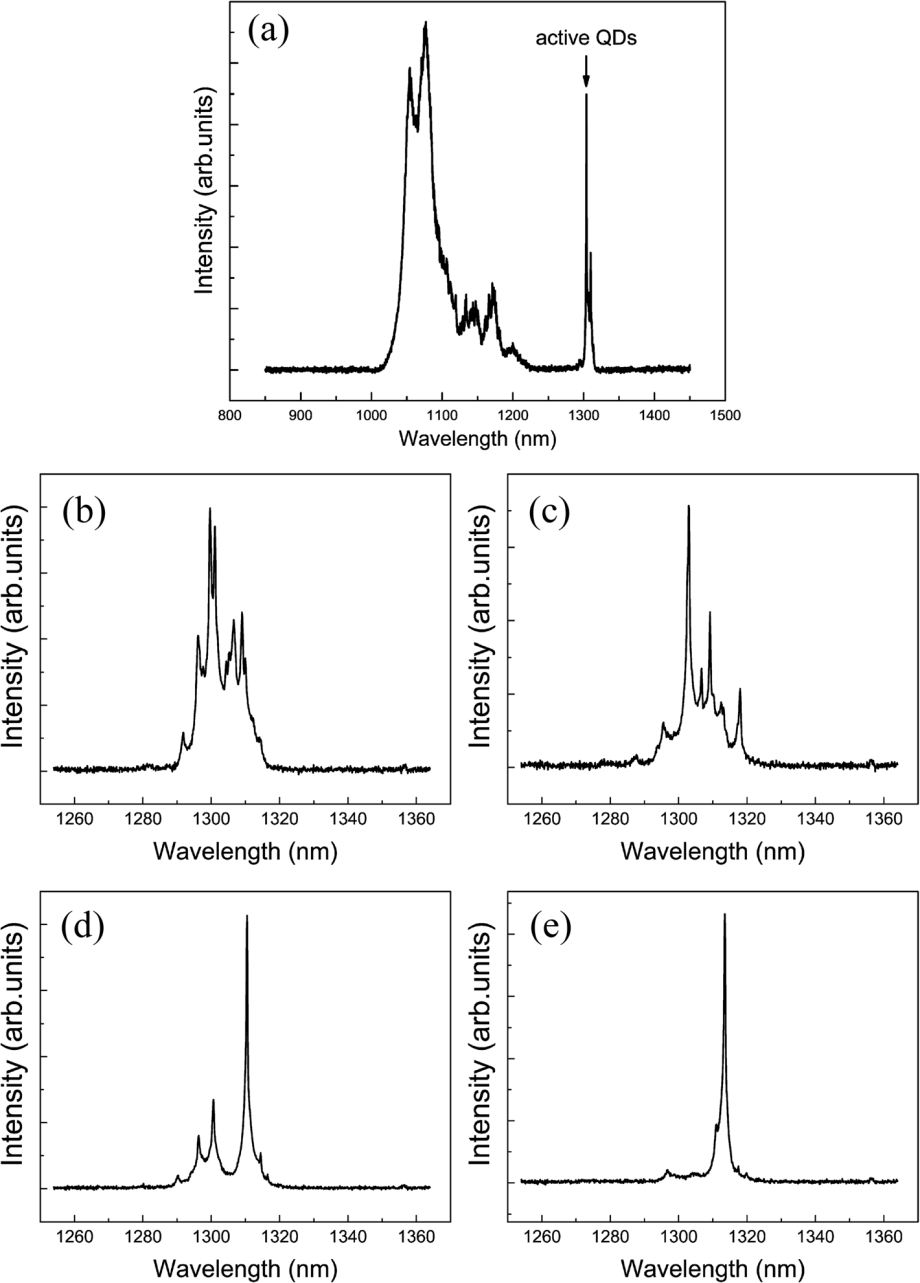
μPL spectra of sample A at 80 K. **a** Full spectrum. **b**–**e** Along the direction of In flux gradient

Figure 3 presents the μPL spectra of sample A along the In flux gradient direction. The sample was cooled at 80 K. In Fig. 3a, some weak and broad peaks appear between 1020 and 1200 nm, which are from QDs in the seed layer and the small QDs in the active layer [14, 37]; the ~1.3-μm emission comes from active large QDs. The trend of μPL spectra is well consistent with that of AFM images in Fig. 2. In Fig. 3b, c, the μPL spectra are envelope profiles, corresponding to high-density active QDs. The multiple peaks in Fig. 3d demonstrate an obvious decreasing of the density of active QDs. In Fig. 3e, the sharp single spectral line suggests single QD formation. The big variation of large QD density and their μPL spectra along the In flux direction suggests that QD formation is very sensitive on the indium coverage. On the other hand, the gradient In flux method ensures a high-repeatability of low-density large QD formation.

**Fig. 4:**
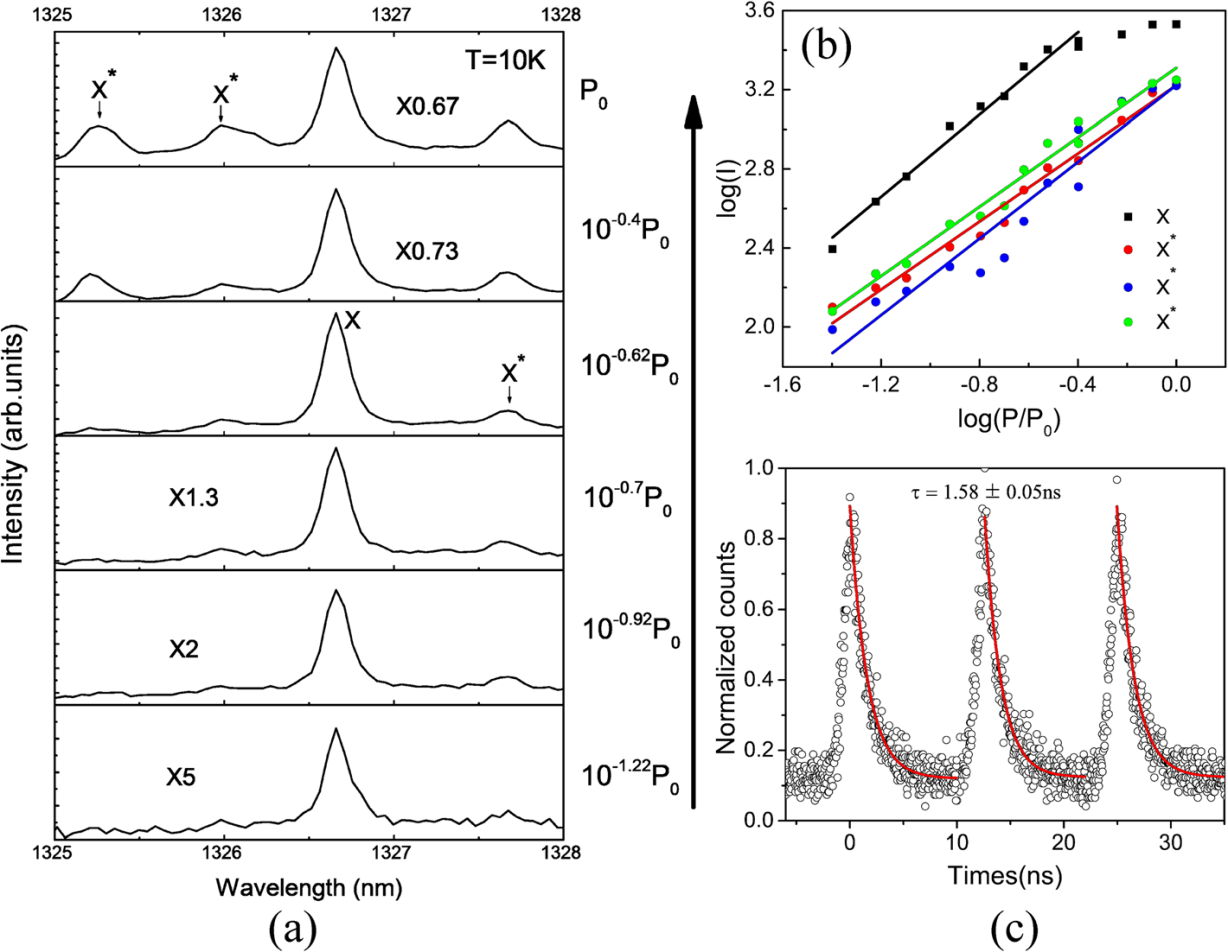
**a** Excitation power-dependent μPL spectra of sample A at 10 K. *X* neutral exciton line, X* charged one. **b** Power dependence of the intensity of exciton lines *X* (*square*) and X* (*circles*). *Colored lines*: linear fitting, showing different slopes. **c** Time-resolved μPL intensity of X line, *red*: exponential decay fitting

To further characterize the spectral lines like in Fig. 3e, power-dependent μPL spectroscopy was performed at 10 K in a cryogen-free bath cryostat [38], as shown in Fig. 4a. The spectrum is dominated by the emission of the X exciton at an emission wavelength of 1326.7 nm. At low excitation power, only the X line appeared, in a narrow full width at half maximum (FWHM) of 111 μeV, limited by the resolution of the measurement system and the spectral diffusion from small QDs [2, 3, 39]. By further increasing the excitation power, several other lines appeared, recognized as X and X* exciton lines [40]. As shown in Fig. 4b, the X line at 1326.7 nm showed a near linear dependence on the excitation power (slope = 1.04). The peaks at 1325.3, 1326, and 1327.7 nm, whose power dependence showed slopes of 0.86, 0.88, and 0.97, respectively, and non-saturation under high-power excitation, came from either charged exciton or exciton recombination in neighboring less efficient QDs; they are named as X*. We used a 80-MHz pulsed semiconductor laser to measure the lifetime of exciton X. In Fig. 4c, three peaks were chosen from the 100-ns time window to show the decay dynamics of a single exciton emission. Acquired from exponential fitting, its emission lifetime is 1.58 ± 0.05 ns on average, typical for the radiative lifetimes of exciton recombination in InAs/GaAs QDs at telecommunication wavelength [1, 41–43].

**Fig. 5:**
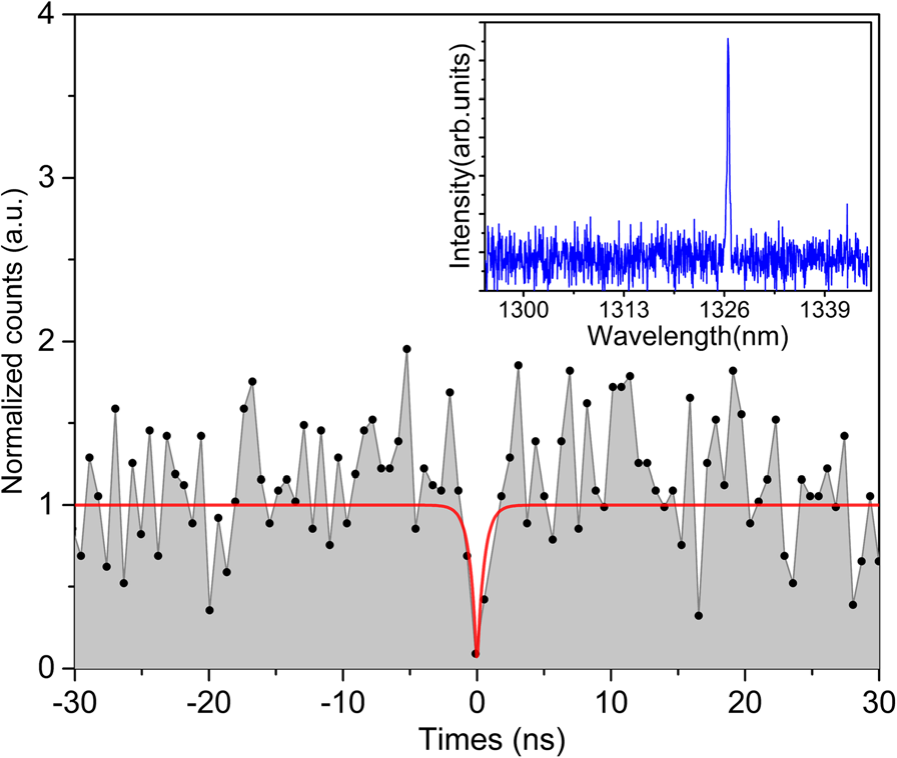
*g* ^2^(*τ*) measurement. This dips to a value of *g* ^2^(0) = 0.088 at 10 K. *Inset*: a typical μPL spectrum after a small grating. The integration time is 8 h

In order to gain insight into the photon statistics of the active QD emission from the BQD material system (i.e., the spectral line in the inset of Fig. 5), we measured the second-order time correlation function *g*^(2)^(*τ*) with a HBT setup under continuous-wave excitation. In order to accumulate the single channel counts in limited time, the time resolution was decreased in the experiment. The correlation dip at zero time delay in Fig. 5 provides evidence of antibunching (i.e., quantum) behavior of the light emission from a large InAs/GaAs QD. The solid curve in red is the fit to the data with the commonly accepted formula [44]. The antibunching dip below 0.5 suggests single-photon emission from single active QD. All the above results prove that the combination of a subcritical InAs coverage and a gradual variation of indium flux is convenient and efficient to obtain low-density and high-quality active QDs from the BQD structure in telecommunication band.

## Conclusions

In summary, MBE growth of low-density active QDs is achieved by using a subcritical InAs deposition technology in seed layer and gradual variation of the indium flux. The AFM results show clear transitions from high-density active QDs to low-density ones. As confirmed by μPL spectroscopy, single active QD was obtained at telecommunication wavelength in the low-density region. Its emission lifetime is about 1.5 ns. The power-dependent μPL spectra suggest single exciton states. The second-order autocorrelation measurement with SNSPD yielded *g*^(2)^(*τ* = 0) < 0.5, demonstrating single-photon emission. In our later experiment, the brightness of single active QD can be well improved by optimizing the growth parameter, i.e., such as growth temperature in active layer. As a novel material system potential for single-photon source in telecommunication wavelength, BQDs are expected to be further optimized for high-efficient emission.

## Abbreviations

QD: Quantum dot
PL: Photoluminescence
*θ*_c_: Critical coverage
SPS: Single-photon sources
DBRs: Distributed Bragg reflectors
DWELL: Dot-in-well
BQD: Bilayer quantum dot
SNSPD: Superconducting nanowire single-photon detector
AFM: Atomic force microscope
HBT: Hanbury-Brown-Twiss
MBE: Molecular beam epitaxy
SQD: Sacrificed InAs quantum dot
RHEED: Reflection high-energy electron-diffraction
SRL: Strain-reducing layer
μPL: Micro-PL
TCSPC: Time-correlated single photon counting
FWHM: Full width at half maximum
